# Dynamics of CD4 T Cell and Antibody Responses in COVID-19 Patients With Different Disease Severity

**DOI:** 10.3389/fmed.2020.592629

**Published:** 2020-11-11

**Authors:** Maximilian Koblischke, Marianna T. Traugott, Iris Medits, Felicia S. Spitzer, Alexander Zoufaly, Lukas Weseslindtner, Cara Simonitsch, Tamara Seitz, Wolfgang Hoepler, Elisabeth Puchhammer-Stöckl, Stephan W. Aberle, Manuela Födinger, Andreas Bergthaler, Michael Kundi, Franz X. Heinz, Karin Stiasny, Judith H. Aberle

**Affiliations:** ^1^Center for Virology, Medical University of Vienna, Vienna, Austria; ^2^Department of Medicine IV, Clinic Favoriten, Vienna Healthcare Group, Vienna, Austria; ^3^Institute of Laboratory Diagnostics, Clinic Favoriten, Vienna Healthcare Group, Vienna, Austria; ^4^Medical Faculty, Sigmund Freud Private University, Vienna, Austria; ^5^Research Center for Molecular Medicine of the Austrian Academy of Sciences, Vienna, Austria; ^6^Center for Public Health, Medical University of Vienna, Vienna, Austria

**Keywords:** SARS-CoV-2, COVID-19 patients, adaptive immunity, SARS-CoV-2-specific antibodies, SARS-CoV-2-specific T cells

## Abstract

Disease caused by the severe acute respiratory syndrome coronavirus 2 (SARS-CoV-2) ranges from mild illness to severe respiratory disease and death. In this study, we determined the kinetics of viral loads, antibody responses (IgM, IgG, neutralization) and SARS-CoV-2-specific CD4 T cells by quantifying these parameters in 435 serial respiratory and blood samples collected from a cohort of 29 COVID-19 patients with either moderate or severe disease during the whole period of hospitalization or until death. Remarkably, there was no significant difference in the kinetics and plateau levels of neutralizing antibodies among the groups with different disease severity. In contrast, the dynamics of specific CD4 T cell responses differed considerably, but all patients with moderate or severe disease developed robust SARS-CoV-2-specific responses. Of note, none of the patients had detectable cross-reactive CD4 T cells in the first week after symptom onset, which have been described in 20–50% of unexposed individuals. Our data thus provide novel insights into the kinetics of antibody and CD4 T cell responses as well as viral loads that are key to understanding the role of adaptive immunity in combating the virus during acute infection and provide leads for the timing of immune therapies for COVID-19.

## Introduction

Severe acute respiratory syndrome coronavirus 2 (SARS-CoV-2) has recently emerged as a new human-to-human transmissible pathogen, causing a pandemic with serious global health consequences. Most infected patients present with mild-to-moderate symptoms and approximately 20% develop severe disease (1). Older people as well as persons with underlying chronic diseases appear to be predisposed to a poor clinical outcome, and male patients have a greater risk of death (2–4). As of June 30, 2020, the World Health Organization (WHO) reported 10.2 million confirmed cases of coronavirus disease (COVID-19), including 503.862 deaths.

SARS-CoV-2 is a lipid-enveloped virus with a positive-stranded RNA genome and four structural proteins (spike glycoprotein, S; envelope protein, E; membrane protein, M; nucleocapsid protein, N). The target of neutralizing antibodies (nAbs) is the S protein, forming prominent projections at the virus surface and mediating viral entry functions. S-specific antibodies directed to both sub-units of S (S1, S2) that prevent these functions can therefore inhibit entry of coronaviruses into cells and potentially protect from disease (5, 6). Passive immunization with convalescent plasma containing such antibodies or strongly neutralizing monoclonal antibodies (mabs) are pursued as a therapeutic option for severe cases [reviewed in (7)]. In addition, most of the current efforts of developing vaccines rely on the use of the S protein as an immunogen.

Infection with SARS-CoV-2 activates innate and adaptive immune responses, including the induction of virus-specific T and B cells, but dysfunctional immune responses, such as inflammatory cytokine storms, are probably associated with the severity of COVID-19 [reviewed in (8)]. CD4 T cells play essential roles in coordinating immune responses via the help to B cells for nAb production. They also promote effector activity of CD8 T cells and the establishment of B and T cell memory (9). SARS-CoV-2-specific CD4 T cells produce IL-2 and IFN-γ, suggesting that COVID-19-recovered individuals exhibit a TH1 cell response (10–12).

Experimental data obtained in non-human primate models indicate that pre-existing virus-specific nAbs and T cells can mediate protection against virus challenge (13, 14). It is unknown, however, how the time course of nAbs as well as T cells correlate with virus clearance and to which extent adaptive immune responses contribute to resolution of disease in the course of infection. We addressed these questions in a comprehensive study of three well-characterized groups of COVID-19 patients with different disease outcomes (moderate, severe, deceased) by quantifying virus loads, Ab responses as well as CD4 T cell responses over the entire time of hospitalization. The goal of this study was to analyze the kinetics of viral load and SARS-CoV-2-specific immune responses. We found that viral loads declined significantly faster in patients with less severe disease, but all patients developed comparable levels of neutralizing antibodies with similar kinetics. In contrast to the antibody response, the dynamics of specific CD4 T cell responses differed considerably, but all patients with moderate or severe disease developed robust antiviral responses.

## Materials and Methods

### Study Cohort

Human blood samples from all patients have been collected under the approval of the Ethics committee of the Medical University of Vienna, Austria (EK 2283/2019). All patients provided written informed consent. The use of anonymized healthy control samples for the validation of serological assays has been approved by the Ethics committee of the Medical University of Vienna, Austria (EK 2156/2019). Between March, 11, 2020 and April, 14, 2020, 29 patients with blood samples available for 14 consecutive days or longer after symptom onset were included (Table 1). The median interval between symptom onset and collection of first blood sample collection was 7 days (IQR 4–11). Of the 29 patients, 13 had moderate disease, requiring low-flow oxygen and were admitted to the normal ward (NW; group 1), nine were severe cases, of whom all required supplemental oxygen (high-flow nasal cannula, non-invasive ventilation, or invasive ventilation), were admitted to the intensive care unit (ICU) and survived (group 2), and seven patients (4 ICU, 3 NW) deceased (group 3). Antiviral treatment included remdesivir, lopinavir/ritonavir, hydroxychloroquine, or human recombinant soluble angiotensin converting enzyme-2 (Supplementary Table 1).

**Table 1:** Demographic data and comorbidities among groups of patients with COVID-19.

	**Group 1 moderate disease (*n* = 13)**	**Group 2 severe disease (*n* = 9)**	**Group 3 deceased (*n* = 7)**	***P*-value**
Age, years	71.9 (29-98)	56.6 (12-77)	77.5 (63–84)	0.025
**SEX**				
Female	9 (69%)	4 (44%)	2 (22%)	0.19
Male	4 (31%)	5 (56%)	7 (78%)	0.19
**CHRONIC COMORBIDITIES**				
Hypertension	4 (31%)	3 (33%)	5 (56%)	0.18
Chronic lung disease	0 (0%)	1 (11%)	1 (11%)	0.41
Diabetes	2 (15%)	2 (22%)	2 (22%)	0.78

*Data are median (range) or numbers (%). P-values derived from Mann Whitney U-test for continuous variables and Fishers exact test for categorical variables*.

### Detection of SARS-CoV-2 RNA

Viral RNA load was determined in endotracheal aspirates (if available in ICU patients) and nasopharyngeal swabs. Briefly, SARS-CoV-2 RNA was extracted from respiratory specimens using NucliSENS easyMAG extractor (BioMérieux, Marcy l'Etoile, France). SARS-CoV-2 real-time TaqMan PCR was performed with WHO recommended primers and probe located in the E-gene, as described previously (15).

### SARS-CoV-2 Virus Isolation

The SARS-CoV-2 strain was isolated from a nasopharyngeal swab from a COVID-19 patient. Vero E6 cells (ATCC® CRL-1586) were infected with the specimen and incubated at 37°C until a cytopathic effect (CPE) occurred. Cell culture supernatant (SN) was harvested and the presence of SARS-CoV-2 was confirmed by PCR. The SN was negative for other human coronaviruses, rhinovirus, metapneumovirus, parainfluenzavirus, influenza A/B viruses, respiratory syncytial virus as well as enteroviruses. The virus isolate was then passaged two more times in Vero E6 cells. The sequence was determined by next generation sequencing and uploaded to the GISAID database (EPI_ISL_438123/hCoV-19/Austria/CeMM0360/2020).

### SARS-CoV-2 Neutralization Test (NT)

Two-fold serial dilutions of heat-inactivated serum or plasma samples were incubated with 50–100 TCID_50_ SARS-CoV-2 for 1 h at 37°C before the mixture was added to Vero E6 cell monolayers (starting dilution of samples 1:10). Incubation was continued for 2–3 days. NT titers were expressed as the reciprocal of the serum dilution required for 100% protection against virus-induced cytopathic effects. NT titers ≥10 were considered positive. For two initially seropositive cases (nAb titers ≥240) with unknown disease onset, the earliest time point of symptom onset was set, assuming that the time to seroconversion was 10 days. Two negative (historical) and three positive (PCR-confirmed patients, 10–14 days after symptom onset) serum samples were included in each assay as controls. The NT was validated with 45 serum samples from healthy controls, including five samples with a prior PCR-confirmed infection with other human coronaviruses (HCoV-OC43 or HCoV-229E, 154–441 days after disease), which all yielded a negative result (NT titer <10).

### Generation of the Recombinant SARS-CoV-2 Spike Protein

The coronavirus spike ectodomain of SARS-CoV-2 (strain Wuhan-Hu-1; residues 1–1213; GenBank: QHD43416.1) was expressed transiently in COS-1 cells (ATCC® CRL-1650) with a C-terminal trimerization motif and a strep-tag using the pCAGGS expression plasmid, kindly provided by Berend Jan Bosch (16, 17). COS-1 cells were electroporated with 5 μg DNA using a Bio-Rad GenePulser apparatus (settings: 1.5 kV, 25 μF, infinity) and were grown for 20–22 h in Dulbecco's modified eagle's medium (DMEM), supplemented with 10% fetal calf serum (FCS) and 1% Penicillin-Streptomycin-Glutamine (both from Gibco). The medium was then replaced with DMEM containing 2% FCS and 25 mM HEPES (Gibco). Incubation was continued for another 72 h. Ninety-six hours after electroporation the supernatant (SN) was harvested and cleared by centrifugation (10,000 rpm; 30 min; 4°C: Beckmann JA 14). To confirm the presence of the strep-tagged ectodomain of the spike, serial dilutions of the SN were added to Strep-Tactin coated microplates (IBA GmbH, Göttingen, Germany) and were incubated for 1 h at 37°C in phosphate-buffered saline (PBS) pH 7.4, 2% sheep serum, 2% Tween 20. A rabbit mab recognizing the S1 subunit of SARS-CoV-2 (Sino Biologicals, Spike S1 Antibody, Rabbit mab, # number 40150-R007) was then added and incubated for 45 min at 37°C. Bound mab was detected with DAR-HRP (Anti-rabbit IgG, horseradish peroxidase (HRP)-linked species-specific whole antibody from donkey, GE Healthcare, # NA 934).

### SARS-CoV-2 IgM and IgG ELISA

COS-1 SN containing the strep-tagged spike protein was diluted 1:3 in PBS pH 7.4, 2% sheep serum, 2% Tween 20 and was added to Strep-Tactin coated microplates (IBA GmbH, Göttingen, Germany) that were blocked for 30 min with 1% bovine serum albumin (BSA) in PBS pH 7.4. Antigen incubation was carried out for 60 min at 37°C. Serial dilutions of human serum or plasma samples (starting dilution 1:100) were added and incubated for 45 min at 37°C. In the case of the IgM ELISA, samples were pre-incubated with rheumatoid-factor-IgG-absorbent (RF absorbent, Siemens Healthcare Diagnostics GmbH, # OUCG15/10446434). Bound human antibodies were detected either with goat anti-human IgM or IgG labeled with HRP (Thermo Fisher Scientific: Goat anti-Human IgM Secondary Antibody, HRP, # 31415. Goat Anti-Human IgG (H+L) Cross-Adsorbed Secondary Antibody, HRP, # 31412). Absorbance was measured at 450 nm. Titers were determined by curve fitting with a four-parameter logistic regression using GraphPad Prism 8 (GraphPad Software Inc.). A positive control serum was included in each test. This control serum was obtained from a COVID-19 patient (16 days after disease onset) with an NT titer of 960. For cut-off determination, we used 30 plasma samples from healthy blood donors. The cut-off for titer determinations was set as the mean absorbance value from these negative controls at a 1:100 dilution plus three standard deviations.

### Preparation of Blood Samples

Peripheral blood mononuclear cells (PBMCs) were separated from whole-blood samples using Ficoll-Paque Plus™ (GE Healthcare) and were cryopreserved in liquid nitrogen, as previously described (18). PBMCs were thawed and depleted of CD8-positive cells using magnetic beads coupled with anti-CD8 antibody and LD columns (Miltenyi Biotec GmbH, Germany), as previously described (19). The depleted PBMCs were incubated overnight in serum-free medium (AIM-V; Gibco) at 37°C in 5% CO_2_. For use in ELISpot assays, cells were resuspended at a final concentration of 2 × 10^6^ cells/ ml in AIM-V. The purity and viability of CD8-depleted PBMCs in each sample was assessed using anti-CD8-APC, anti-CD3-PE, anti-CD4-PacificBlue™, and 7-aminoactinomycin D (BD Bioscience) and flow cytometry (18). Purity of CD8-depleted PBMCs was usually >99%. Plasma and serum samples were stored at −20°C.

### Peptides

For T cell stimulation, four PepMix^TM^ SARS-CoV-2 peptide pools (product codes: PM-WCPV-VEMP, PM-WCPV-VME, PM-WCPV-S, and PM-WCPV-NCAP) were purchased from JPT (Berlin, Germany). The pools comprise 15 mer peptides overlapping by 11 amino acids and cover the entire sequences of the SARS-CoV-2 structural proteins: envelope (E), membrane (M), spike (S), and nucleoprotein (N). The S pool is composed of two sub-pools S1 (aa 1-643) and S2 (aa 633-1273). Peptides were dissolved in dimethyl sulfoxide and diluted in AIM-V medium for use in ELISpot assays.

### IFN-γ ELISpot Assay

IFN-γ ELISpot assays were performed as previously described (12, 13). Briefly, plates (MSIPS4W10, Merck-Millipore) were coated with 1.5 μg anti-IFN-γ antibody (3420-3-1000, Mabtech) per well. For blocking, PBS/5% BSA (11930, Serva) was used. The CD8-depleted PBMCs of COVID-19 patients (1 × 10^5^ cells/well) were incubated at 37°C and 5% CO_2_ for about 45 h with SARS-CoV-2 peptides (2 μg/ml; duplicates), AIM-V medium (negative control; 2–4 wells), or leucoagglutinin (PHA-L; L4144, Sigma; 0.5 μg/ml; positive control). After washing, spots were developed with 0.1 μg biotin-conjugated anti-IFN-γ antibody (3420-6-250, Mabtech), streptavidin-coupled alkaline phosphatase (ALP; 3310-10, Mabtech; 1:1,000) and 5-bromo-4-chloro-3-indolyl phosphate/nitro blue tetrazolium (BCIP/NBT; B5655, Sigma). The number of spots was evaluated using a Bio-Sys Bioreader 5000 Pro-S/BR177. Spots were counted using automatically calculated spot-size thresholds (upper and lower gates) to distinguish spots produced by antigen-specific T cells from cell clusters and from non-specific background spots with Bioreader v 10 software. Responses to SARS-CoV-2 peptide pools were defined positive if at least two-fold above the mean +3 SD of spots from 5 healthy controls who tested negative for coronavirus S-specific IgG (≥50 spots). The ELISpot assay was validated by comparing IFN-γ responses between undepleted PBMC controls, CD4-depleted, and CD8-depleted PBMCs, as described previously (20). FACS analysis revealed that cell depletion by magnetic bead separation was complete (Supplementary Figure 1). The sums of responses from CD4- and CD8-depleted fractions were comparable to PBMC controls.

### Statistics

**Figure 1:**
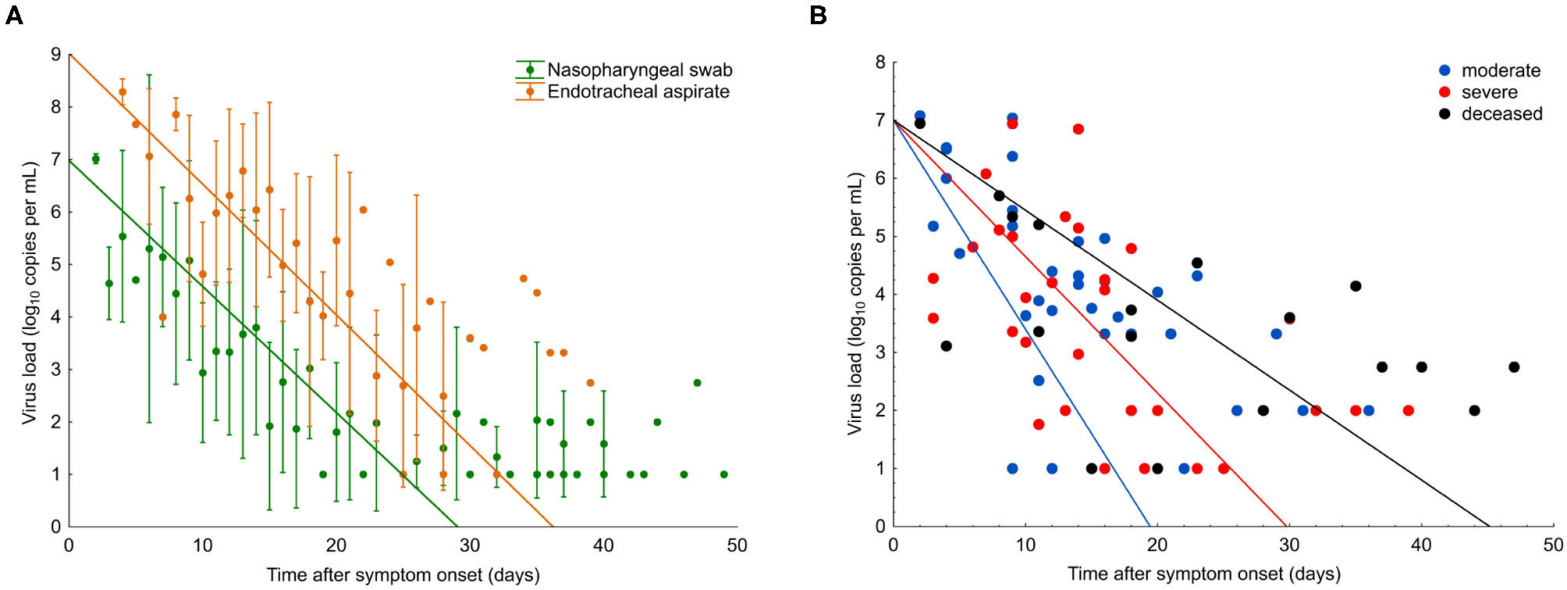
SARS-CoV-2 viral load in COVID-19 patients with different disease severity. **(A)** Viral load from nasopharyngeal swabs (green) and endotracheal aspirates (orange). Data points are mean; error bars indicate SD; slopes represent best fit. **(B)** Viral load from nasopharyngeal swabs from all patients (*n* = 29). Data points indicate viral load in individual samples; slopes represent viral RNA decline in patient groups, as assessed by Generalized Estimation Equations (GEE) applying an unstructured correlation matrix. Group one, moderate (blue); group two, severe (red), and group three, deceased (black).

Statistical analysis was performed using SPSS version 26.0 and Prism version 5.0. Combined correlation coefficient was calculated by using Fisher's z′ transformation and averaging over patients to assess the relation between virus loads from nasopharyngeal swabs and endotracheal aspirates. Decline of virus loads in nasopharyngeal swabs and endotracheal aspirates was assessed by Generalized Estimation Equations (GEE) model applying an unstructured correlation matrix. This analysis was restricted to the first 30 days after disease onset and only the first negative test result was included. First, a model with homogeneous slope was fit (Figure 1A), however, a model with heterogeneous slope fit the data better according to Akaike's criterion and was applied to compare groups with respect to decline behavior. The GEE model Walsh chi^2^ test was conducted to analyse variables (age, sex or comorbidities) potentially associated with differences in vRNA decline, IgG, IgM, NT titer, and CD4 T cell response. Pearson's correlation analyses was performed to assess the relationship between nAb titers or CD4 T cell levels and anti-S IgM, IgG, and between viral RNA and nAb titers or CD4 T cell levels. Dunn's multiple comparisons following a Kruskal Wallis test were performed for analysis of IFN-γ ELISpot assays. Statistical significance was determined as *P* < 0.05 (^*^P < 0.05, ^**^P < 0.01, and ^***^P < 0.001).

## Results

### Patients and Clinical Outcome of Disease

We analyzed viral loads, virus-specific antibody, and CD4 T cell responses in 29 COVID-19 patients over the entire period of their hospitalization. The basic characteristics of these patients are displayed in Table 1 and more specific information (including therapies) are shown in Supplementary Table 1. The patients were divided into three groups, according to disease outcome, classified as “moderate disease,” “severe disease,” and “deceased.”

Thirteen cases had moderate disease, but still required hospitalization and were admitted to the normal ward (NW; group 1), nine were severe, of whom all were admitted to intensive care unit (ICU) and survived (group 2), and seven patients (4 ICU, 3 NW) deceased (group 3). The median age of all patients was 71.9 years (range 29–98).

### Viral RNA Load

For the comparison of viral RNA (vRNA) loads over time in the three different patient groups, we analyzed 271 respiratory specimens, including 203 nasopharyngeal swabs and 68 endotracheal aspirates collected between 2 and 49 days after symptom onset. In nasopharyngeal swabs, the overall median viral load at the time point of presentation was 5.1 log_10_ copies/ml (interquartile range, IQR 4.0–6.5) and continuously declined over the course of disease (Figure 1A). Endotracheal aspirates (collected from 10 patients, six from group 2, and four from group 3) had, on average, 100 times higher copy numbers/ml than nasopharyngeal swabs (Figure 1A). A significant correlation (*r* = 0.71, *p* < 0.01) was found between the vRNA copy numbers in the two materials during the time course of disease (Figure 1A).

In the first samples, collected within a median of 8 days after symptom onset (IQR 4–10), viral loads were not significantly different between the three patient groups (*p* = 0.15). The decline of vRNA, however, was significantly slower in groups 2 and 3 than in group 1 (*p* < 0.01), as determined by a generalized estimating equation model (Figure 1B). Significantly more patients in groups 2 (7/9) and 3 (4/7) received antiviral treatment than group 1 (2/13) (*p* = 0.0115). There was no significant difference in vRNA decline among the patients who received different antiviral therapies, including remdesivir (vRNA halflife, 4.0; IQR 2.6–8.4), lopinavir/ritonavir (vRNA halflife, 3.3; IQR 2.5–4.9), and hydroxychloroquine (vRNA halflife, 4.0; IQR 2.6–8.4). Analysis of vRNA loads by age, sex or chronic comorbidities in generalized estimating equation model, Walsh chi^2^ tests revealed that vRNA decline was significantly slower in patients older than 65 years (*p* = 0.024) and in patients with chronic lung disease (*p* = 0.03), whereas no effect was seen with hypertension (*p* = 0.228) or diabetes (*p* = 0.900).

### Neutralizing Antibody Titers and Correlation With Anti-S IgM and IgG Titers

**Figure 2:**
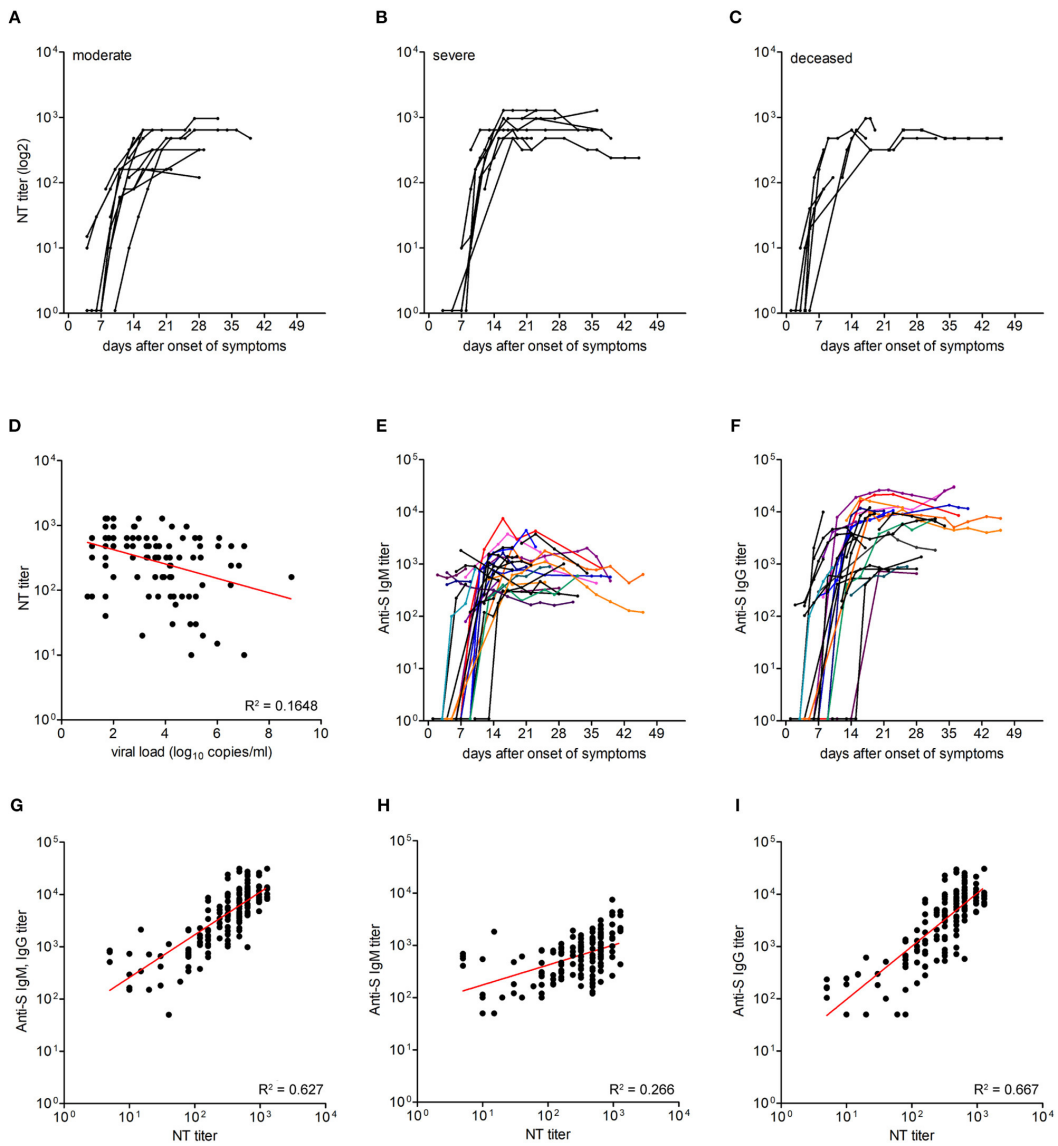
Titers of neutralizing antibodies and correlation with viral load and IgM and IgG titers against the spike protein. **(A–C)** Neutralizing antibody titers in moderate (*n* = 13), severe (*n* = 9), and deceased (*n* = 7) patients. **(D)** Correlation between neutralizing antibody titers and virus RNA loads was assessed using Pearson correlation. **(E,F)** IgM and IgG ELISA titers against the spike protein in 29 patients. Each line represents an individual patient. **(G–I)** Correlation between neutralizing antibody titers and anti-S IgM and IgG ELISA titers, anti-S IgM ELISA titers and anti-S IgG ELISA titers, as assessed by Pearson correlation.

To assess whether there was a correlation between the extent of viral loads as well as disease outcomes and a specific humoral immune response, we first quantified neutralizing antibodies in 161 sequential serum/plasma samples from the three patient groups (median 5 serum specimens per patient). The results are shown in Figures 2A–C. In the first samples (median day 7 after onset of symptoms, IQR, 4–11), 16 patients already had detectable nAbs, and 13 patients were seronegative. No association was seen between seropositivity at presentation and severity of illness (Chi square = 2.1; *p* = 0.4). In the course of disease, all patients developed nAbs, which were negatively correlated with vRNA loads (Pearson *r* –0.446; *p* < 0.0001; Figure 2D). The titers showed a steep rise between days 6–11 and reached a plateau between days 15 and 22 after symptom onset. The plateau titers were quite high (median, 640; IQR 440–720), and there was no significant difference of these titers among the three patient groups (*p* = 0.32, Kruskal-Wallis test).

To determine the correlation between neutralizing antibody titers and IgM and IgG responses to the spike (S) protein, we performed corresponding ELISAs (using the whole ectodomain of S as an antigen) with sequential samples (*n* = 158) of the 29 patients (Figures 2E,F). Fourteen patients were already either IgM, IgG or IgM, and IgG positive in the first samples obtained between 2 and 14 days after symptom onset, and 11 of these samples were also NT positive. Seroconversion for anti-S IgM or IgG was observed on days 11 (IQR 9–14) and 12 (IQR 10–18) after symptom onset, respectively. In the assays used, plateau titers of IgM (log 3.1, IQR 2.8–3.4) were similar to NT titers (log 2.8, IQR 2.6–2.9), and IgG were about ten-fold higher than IgM titers (log 4.0, IQR 3.7–4.2). There was a positive correlation between total anti-S Ab (IgM and IgG) titers and nAb titers (Pearson *r* 0.792, 95% CI 0.73–0.84; *R*^2^ 0.627; *p* < 0.0001; Figure 2G). The correlation between anti-S IgG titers and nAb titers (Pearson *r* 0.817, 95% CI 0.76–0.86; *R*^2^ 0.667; *p* < 0.0001) was stronger than between anti-S IgM titers and nAb titers (Pearson *r* 0.516, 95% CI 0.39–0.62; *R*^2^ 0.266; *p* < 0.0001; Figures 2H,I).

### SARS-CoV-2 Specific CD4 T Cell Responses

To investigate whether the extent and time course of specific CD4 T cell responses correlated with disease outcome, we analyzed peripheral blood mononuclear cells (PBMCs) from 21 patients (eight from group 1; eight from group 2; five from group 3). For this purpose, CD8-positive cells were depleted from PBMCs, and CD4 T cell responses were quantified by IFN-γ enzyme-linked immunosorbent spot (ELISpot) assays. IFN-γ ELISpot assays were performed using pools of peptides covering the entire sequences of all four viral structural proteins S, M, N, and E. Sequential samples were available from 17 of the 21 patients.

**Figure 3:**
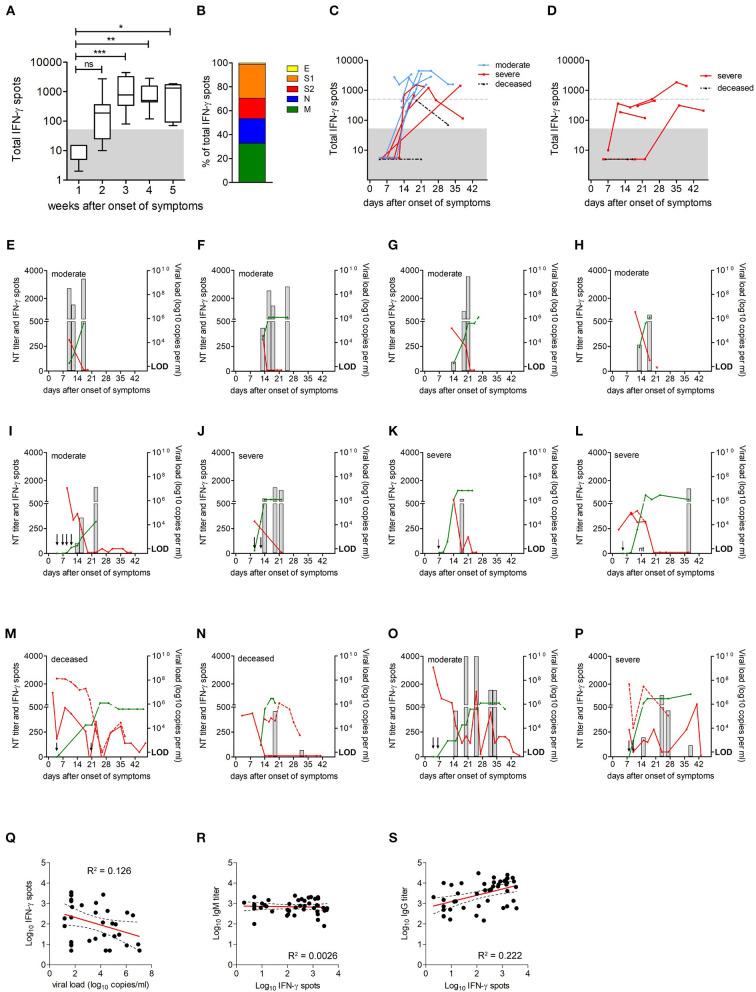
Extent of SARS-CoV-2-specific CD4 T cell responses over time. **(A)** Extent of CD4 T cell responses to the four SARS-CoV-2 structural proteins, as determined by IFN-γ ELISpot assays (*n* = 21); data are presented as box and whiskers plots, with bounds from 25th to 75th percentile,plots, with bounds from 25th to 75th percentile, median line, and whiskers ranging from minimum to maximum of total IFN-γ spots. Significance was determined by Kruskal Wallis test, *P < 0.05, **P < 0.01, ***P < 0.001. Area below cut off in IFN-γ ELISpot assay (<50 spots per 106 PBMCs) is shaded gray. **(B)** Percentage of spots contributed by S1, S2, M, N, and E. **(C)** Kinetics of CD4 T cell responses in patients with moderate or severe disease and in deceased patients; group one, moderate (blue circles); group two, severe (red squares) and group three, deceased (black triangles). **(D)** Kinetics of CD4 T cell responses in patients with corticosteroid therapy (*n* = 5); group two, severe (red squares) and group three, deceased (black triangles). Dotted gray lines indicate 500 spots (i.e., 10 times the cut-off of the ELISpot assay). Area below cut off in IFN-γ ELISpot assay (<50 spots per 10^6^ PBMCs) is shaded gray. **(E–P)** CD4 T cell responses (gray columns), neutralizing antibody titers (green lines), and virus loads in nasopharyngeal swabs (red lines) or endotracheal aspirates (dotted red line) in individual patients. Arrows indicate time points of ELISpot assays with no detectable CD4 T cell reactivity; red star indicates discharge, negative PCR result was not obtained; nt, not tested; LOD, limit of detection. **(Q–S)** Correlations between virus-specific CD4 T cell levels and vRNA loads, anti-S IgG, or IgM ELISA titers were assessed using Pearson correlation.

As shown in Figure 3A, no specific CD4 T cell reactivity was detectable in the first week after symptom onset. After this initial delay, all, except two deceased patients developed detectable antiviral CD4 T cell responses. Overall, the magnitude of CD4 T cell responses increased until week 3 after symptom onset (Figure 3A). The contribution of viral proteins to overall CD4 T cell responses is displayed in Figure 3B and shows that S (including S1 and S2) and M dominated the response, contributing 45% and 33% to measured reactivities, respectively. The contribution by N was somewhat lower (21%) and that of E was only marginal (1%), corresponding to the amounts of the proteins in the virus particle (21).

Since corticosteroid therapy can have a profound T cell suppressive effect (22), the kinetics of CD4 T cell responses from patients with or without corticosteroid therapy were studied separately. The analysis of response kinetics from patients not receiving corticosteroid therapy revealed considerable individual variation, as displayed in Figure 3C. All patients from groups 1 and 2 mounted a robust CD4 T cell response, reaching at least 10 times the cut-off of the ELISpot assay. However, differences were observed with respect to the time point when strong responses became detectable, ranging from 14 to 24 days after symptom onset (Figure 3C). Of the deceased patients, one had no response at days 4 and 21 after symptom onset, and the second patient mounted a low response at day 19, which dropped toward the cut-off at day 32 after symptom onset (Figure 3C). For 4 of the 5 patients who received corticosteroids, no or low antiviral CD4 T cell responses were detected, and one patient mounted a robust response >500 spots, but only 5 weeks (days 35 and 39) after symptom onset (Figure 3D).

We next analyzed the time course of CD4 T cell responses in relation to viral clearance for all patients shown in Figure 3C. The most common pattern in patients from group 1 and group 2 was characterized by a robust CD4 T cell response followed by viral clearance (Figures 3E–L). Of the two deceased patients, one had detectable CD4 T cells after vRNA clearance, and one did not mount a detectable response until week 3 after symptom onset and had several virus rebounds until finally deceased (Figures 3M,N). In accordance with recent studies (12), CD4 T cell levels were negatively correlated with vRNA loads (Pearson *r* −0.3555; *p* = 0.0390; Figure 3Q). In addition, strikingly different patterns were observed in two cases. Specifically, one patient from group 1 and one from group 2 developed strong CD4 T cell responses, but nevertheless had an early virus rebound and prolonged infection (Figures 3O,P). There were no significant differences in CD4 T cell response kinetics in relation to patients sex or age (sex, *p* = 0.469; age >65 years, *p* = 0.943; generalized estimating equation model, Wald chi^2^ test).

Because CD4 T cells play an important role in promoting efficient antibody production through support of antibody class switch and the development of high-affinity antibody-secreting B cells, we correlated CD4 T cell levels with antibody titers. As shown in Figures 3R,S, there was a positive correlation between CD4 T cell levels and anti-S IgG titers (Pearson *r* 0.4714; *p* = 0.0011), whereas no correlation was observed between CD4 T cell responses and anti-S IgM titers (Pearson *r* −0.0511; *p* = 0.7388), indicating that the development of IgG is correlated with the activation of virus-specific CD4 T cells.

## Discussion

In this study, we provide a comprehensive quantitative analysis of the time course of viral loads, neutralizing antibody and CD4 T cell responses in 29 COVID-19 patients with different disease outcomes over the whole period of hospitalization or until death. In line with previous reports [reviewed in (23)], all patients developed high levels of SARS-CoV-2 S-specific antibodies. Remarkably, there was neither a significant difference in the kinetics nor in the plateau levels of nAb responses among the patients with different outcomes, even in those succumbing to the disease, indicating that antibody levels are not predictive for the outcome of the disease. People with an asymptomatic SARS-CoV-2 infection were reported to have lower titers of virus-specific antibodies or were even seronegative compared to patients with severe disease (24, 25), indicating that other arms of the immune system control infection in these people. Challenge studies with non-human primates have demonstrated a protective role of nAbs when present before SARS-CoV-2 infection (13, 14, 26, 27). In acute infection, however, the production of nAbs thus appears to be too late for contributing to virus clearance and/or resolving disease.

The observed kinetics of virus and antibody titers have implications for therapies based on antibodies, administered as either convalescent plasma or mabs [reviewed in (7, 28)]. As already deduced from preliminary trials (28), the success of passive antibody therapy requires a good timing of administration. Our data based on a tight sampling schedule during hospitalization indicate that the therapeutic window is at (or very early after) symptom onset, when virus titers are still high, but Abs are not yet detectable. A further important parameter for convalescent plasma therapy is the use of preparations with confirmed high titers of nAbs, thus probably limiting the donors to people recovered from symptomatic disease. In this respect, it is good news that nAb responses showed an excellent correlation with those obtained in an ELISA using the trimeric ectodomain of S (Figure 2F), in agreement with other studies in which either the whole spike and/or its receptor-binding domain were used (11, 17, 29–32). In some early phase samples, we observed neutralization when IgG were not yet detectable but IgM were already present, indicating that IgM Abs alone can neutralize the virus. Assays detecting S-specific IgG as well as IgM antibodies might thus be valuable surrogate tools for predicting nAb levels of patients in early convalescence.

The picture of antiviral CD4 T cells is more heterogeneous as compared to antibodies, but important features can be discerned. Specific CD4 T cells were not detected in the first week after onset of symptoms, but then increased over time. All patients with moderate and severe disease developed robust antiviral CD4 T cell responses, which were negatively correlated with vRNA loads, consistent with a recent report (12). The data based on multiple sequential samples indicate that the kinetics of the response was highly variable. The measured CD4 T cell activity can therefore be strongly influenced by the timing of sample collection, which points to possible pitfalls that could arise from data collected at single time points only. In one deceased patient, we even did not detect any specific CD4 T cells. The difference in responses might be due to an inflammation-triggered sequestration of antigen-specific cells to the infected tissue, which may eventually reduce detectability in the peripheral blood (33, 34). In our study, we did not observe significant differences in immune response kinetics in relation to age, sex or co-morbidities. However, it is important to note that the sample size in the present study was small, and the combined effects of these factors on immune response kinetics will have to be clarified in larger cohort studies.

Recent data indicate the presence of SARS-CoV-2-reactive T cells in healthy controls, not previously exposed to SARS-CoV-2. These cells could be cross-reactive and the result of previous infection with other human coronaviruses (10, 12, 35, 36). None of the patients in our groups of moderate or severe disease had detectable antiviral CD4 T cells in the samples obtained in the first week, indicating that there was no pre-existing immunity in these cases. Whether the presence of pre-existing cross-reactive CD4 T cells may affect disease outcome and prognosis needs to be addressed in future prospective studies.

In conclusion, our data elucidate the dynamics of adaptive immune responses during the course of hospitalization with moderate or severe COVID-19. Since prolonged virus shedding and virus rebound was observed in patients with moderate and severe disease despite the presence of high titers of neutralizing antibodies and robust CD4 T cell responses, these arms of the immune response do not appear to be able to prevent progression to severe disease. Due to ethical reasons, the blood volume that could be collected for multiple sequential samples at different time points was limited. Thus, we were not able to analyze the kinetics of CD8 T cells or other cytokine-producing CD4 T cell subsets in parallel to Abs, and the possible beneficial or detrimental role of these cells in viral clearance or the pathogenesis of COVID-19 will have to be resolved in future studies. It is likely that the efficient interplay between helper CD4 T cells and B cells to promote the production of high-affinity and potently neutralizing antibodies is essential for inducing post-infection immunity. How long such immunity is maintained and whether sufficiently durable immunity can be induced by active immunization are key questions in the search for an effective vaccine and for understanding the epidemiology of COVID-19 in the future.

## Data Availability Statement

The raw data supporting the conclusions of this article will be made available by the authors, without undue reservation.

## Ethics Statement

The studies involving human participants were reviewed and approved by Ethics committee of the Medical University of Vienna, Austria. The patients/participants provided their written informed consent to participate in this study.

## Author Contributions

JA, KS, and FH: conceptualization and writing. MKo, IM, KS, SA, FS, CS, LW, AB, and MKu: methodology. MKo, JA, KS, MT, IM, and AB: investigation. EP-S, MT, AZ, TS, WH, and MF: resources. JA: funding acquisition. KS and JA: supervision. All authors contributed to the article and approved the submitted version.

## Conflict of Interest

The authors declare that the research was conducted in the absence of any commercial or financial relationships that could be construed as a potential conflict of interest.
