# Supplementary material for: Dynamics of CD4 T Cell and Antibody Responses in COVID-19 Patients With Different Disease Severity
Source: Front Med (Lausanne). 2020 Nov 11;7:592629. doi: 10.3389/fmed.2020.592629 (PMC7686651; doi:10.3389/fmed.2020.592629)
Supplement: Supplementary file 1 [file Data_Sheet_1.PDF]

Supplementary Material

Dynamics of CD4 T cell and antibody responses in COVID-19 patients with different disease severity

Maximilian Koblischke[1#], Marianna T. Traugott[2#], Iris Medits[1#], Felicia S. Spitzer[1], Alexander Zoufaly[2], Lukas Weseslindtner[1], Cara Simonitsch[1], Tamara Seitz[2], Wolfgang Hoepler[2], Elisabeth Puchhammer-Stöckl[1], Stephan W. Aberle[1], Manuela Födinger[3,4], Andreas Bergthaler[5], Michael Kundi[6], Franz X. Heinz[1], Karin Stiasny[1*] and Judith H. Aberle[1*]

[1]Center for Virology, Medical University of Vienna, Vienna, Austria

[2]Department of Medicine IV, Clinic Favoriten, Vienna Healthcare Group, Vienna, Austria

[3]Institute of Laboratory Diagnostics, Clinic Favoriten, Vienna Healthcare Group, Vienna, Austria

[4]Medical Faculty, Sigmund Freud Private University, [5]Research Center for Molecular Medicine of the Austrian Academy of Sciences, Vienna, Austria

[6]Center for Public Health, Medical University of Vienna, Vienna, Austria.

[#]These authors contributed equally to this work

***Correspondence:**

Judith H. Aberle
judith.aberle@meduniwien.ac.at

Karin Stiasny
karin.stiasny@meduniwien.ac.at

Table S1. Demographic data and treatment of patients with COVID-19

Demographic characteristics	
Age, years	71.9 (56.2-78.8)
Sex (male/female)	14 m/15 f
Time from illness onset to collection of first serum sample, days	7 (4-11)
Chronic comorbidities	
Hypertension	12 (41)
Chronic lung disease	2 (6.9)
Diabetes	8 (27.6)
Outcomes	
Time to viral clearance after symptom onset, days	21.5 (17-37)
Time from illness onset to death or discharge, days	26 (12-47)
Treatments	
Hydroxychloroquine	4 (13.8)
Lopinavir/ritonavir	6 (20.7)
Human recombinant soluble ACE2	1 (3.4)
Tozilizumab	2 (6.9)
Corticosteroids	6 (20.7)
High-flow nasal cannula oxygen therapy	4 (13.8)
Non-invasive mechanical ventilation	2 (6.9)
Invasive mechanical ventilation	10 (34.5)

Data are median (IQR) or n (%). ACE2, angiotensin converting enzyme-2

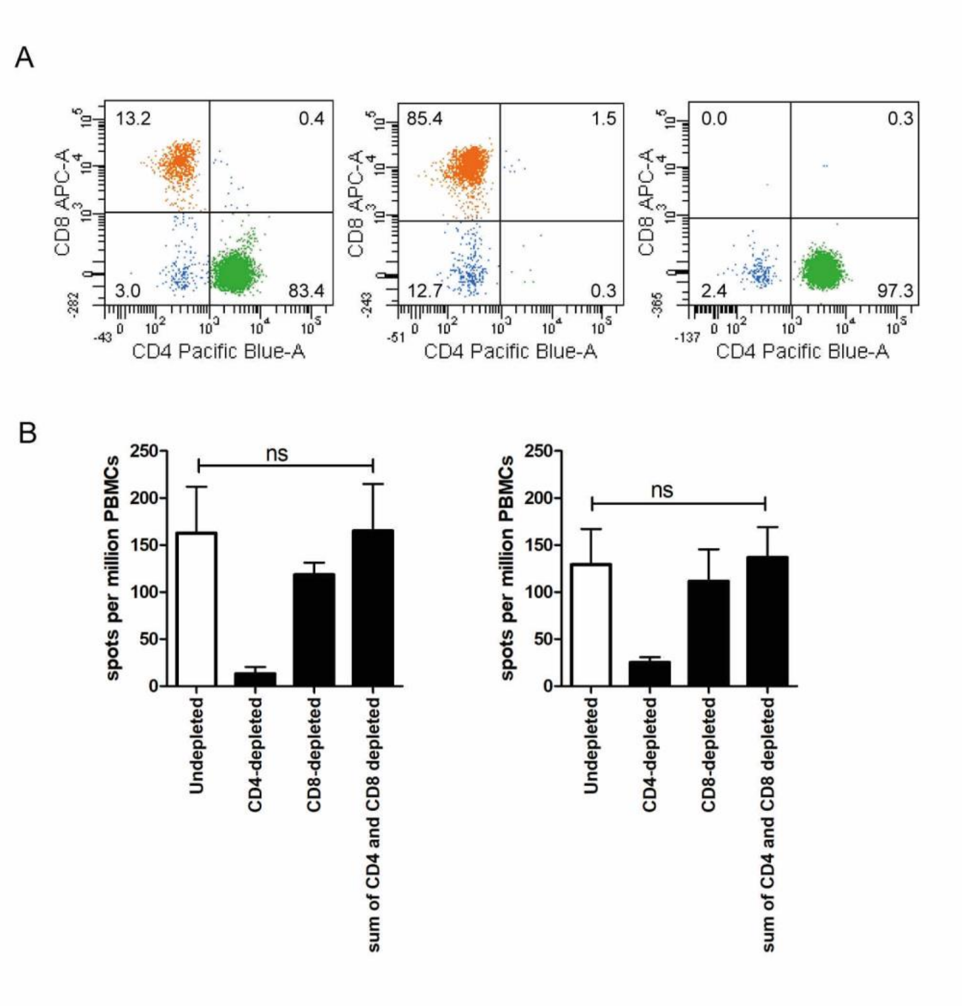

Figure S1. Cell depletion and IFN-γ ELISpot assay. (A) Representative FACS plots of undepleted PBMC controls, CD4-depleted and CD8-depleted PBMCs. (B) IFN-γ ELISpot response in undepleted PBMCs, CD4-depleted and CD8-depleted PBMCs from the same SARS-CoV-2-infected patients after stimulation with peptide pools covering the entire sequences of either M (left) or S1 (right). Each experiment was performed in triplicates.
